# SOX10 Overexpression Enhances the Oligodendrocyte Lineage Commitment of iOPCs In Vitro by Reshaping Their Chromatin Binding Landscape

**DOI:** 10.3390/bioengineering13050500

**Published:** 2026-04-25

**Authors:** Fan Zhang, Zhaoyan Wang, Dou Ye, Jialan Liang, Hui Yang, Suqing Qu, Qian Wang, Zuo Luan

**Affiliations:** 1 Department of Pediatrics, The Sixth Medical Center of PLA General Hospital, Beijing 100048, China; 2 Medical School of Chinese PLA, Beijing 100853, China

**Keywords:** OPC, oligodendrocyte, differentiation, transcription factor, SOX10

## Abstract

Although transplantation of induced oligodendrocyte progenitor cells (iOPCs) is a promising strategy for white matter injury, the therapeutic efficacy of in vitro-generated iOPCs remains limited due to insufficient differentiation potential. Here, we aimed to identify key transcription factors and small-molecule drugs to optimize iOPC quality. Through transcriptome sequencing and bioinformatics analysis, we identified the transcription factor SOX10, which is differentially expressed between endogenous fetal OPCs and exogenous iOPCs. We established lentivirus-mediated *SOX10* overexpression in neural stem cells (NSCs) before iOPC induction and performed cellular assays and multi-omics analysis. Early *SOX10* overexpression reduced cell migration but promoted maturation into oligodendrocytes and suppressed astrocyte differentiation. Multi-omics analyses revealed that *SOX10* overexpression is associated with the extensive redistribution of SOX10 chromatin binding and enrichment of regulatory programs linked to oligodendroglial differentiation, including the activation of the key signaling downstream transcription factors JUN/FOS. Moreover, TSA, Dabrafenib, and Fedratinib effectively upregulated SOX10 and improved iOPC differentiation. This study identifies SOX10 as a core upstream regulator governing the fate of iOPCs, providing a potential strategy for optimizing iOPC induction for future investigation of white matter injury therapy.

## 1. Introduction

OPCs are multipotent progenitor cells with self-renewal capacity that exist in the mammalian central nervous system [1]. Derived from NSCs, they can differentiate directionally into oligodendrocytes (OLs) and participate in myelination, which is fundamental for maintaining normal neural function [2]. The unique biological characteristics of OPCs make them an important part of cell therapeutic approaches for impaired myelination in the central nervous system, such as that occurring in preterm infant white matter injury [3]. At present, there are two main technical approaches for obtaining clinical-grade human OPCs. First, direct isolation from human brain tissue yields cells with high purity and preserves the physiological properties of native cells, but is limited by scarce donor sources and low single-batch yields [4]. Second, iOPCs can be induced from other human cells, including NSCs, embryonic stem cells, and induced pluripotent stem cells. Among these, human NSCs offer the advantages of a short induction period, sufficient cell yield, and low tumorigenic risk [5,6,7].

In the human fetal brain, the earliest detectable OPCs appear in the forebrain at 9 gestational weeks, and the first wave of MBP-expressing myelinating OLs is detected in the thalamus at 18 gestational weeks [8]. Although iOPCs can rapidly differentiate into OLs in vitro, this is largely attributed to the favorable environment provided by nutrient-rich and pro-differentiation factors. OPCs possess bidirectional differentiation potential toward OLs and astrocytes, and the heterogeneity of OPCs from different sources leads to variations in their differentiation potential [9]. To establish an iOPC master cell bank for transplantation therapy, we take fetal brain-derived OPCs as the reference standard. By comparing the transcriptional profiles between iOPCs and endogenous OPCs, we clarify the advantages and limitations of iOPCs and further optimize their seed cell properties on this basis. This study aims to provide experimental evidence and theoretical support for establishing a stable, efficient, and more physiologically relevant iOPC cell bank.

Transcription factors are a class of protein molecules that regulate the selective expression of genes by binding to specific gene regions, and are particularly important for initiating the cell fate determination program that drives stem cell differentiation. For example, LHX2 is a key determinant of cell fate involved in directing the differentiation of radial glial cells into either NSCs or ependymal cells, with changes in its expression directly affecting the differentiation ratio of the two cell lineages [10]. On this basis, we focused on screening SOX10, a key transcription factor capable of enhancing the OL differentiation potential of iOPCs. By performing *SOX10* overexpression experiments in iOPCs combined with cell biological identification assays, we clarified its regulatory effect on the differentiation potential of iOPCs. Meanwhile, to further optimize the iOPC induction medium, we carried out small-molecule drug screening to identify small-molecule compounds that can effectively promote the expression of the target transcription factor. To explore the regulatory mechanisms of transcription factors, we performed an integrated analysis using three sequencing technologies: ATAC-seq, ChIP-seq, and RNA-seq. This work aims to reveal a putative gene regulatory pathway—“chromatin accessibility–transcription factor binding–gene transcription”—laying a foundation for further dissecting the molecular mechanisms by which transcription factors regulate iOPC differentiation.

## 2. Materials and Methods

### 2.1. Cell Culture

The stem cells used in this study were derived from a cell line established by isolating NSCs from aborted human fetal brains in the Pediatric Laboratory of the Sixth Medical Center of Chinese People’s Liberation Army General Hospital.

NSCs were suspension-cultured as spheres in T75 flasks (Corning Incorporated, Corning, NY, USA) and passaged every 7–10 days. The NSC culture medium was prepared by mixing DMEM and F-12 medium at a volume ratio of 3:1, supplemented with the following components: 1% GlutaMAX™ supplement, 15 mM HEPES, 0.15% D-glucose, 100 µg/mL transferrin, 20 nM progesterone, 60 µM putrescine, 30 nM sodium selenite, 5 µg/mL insulin, 5 µg/mL heparin, 20 ng/mL bFGF, 20 ng/mL EGF, 10 ng/mL LIF, and 1% P/S.

Prior to the induction of iOPCs, T75 flasks were coated with PBS containing 10 µg/mL fibronectin and 5 µg/mL laminin for 1 h. The OPC medium was DMEM/F-12 supplemented with 2% B-27™ supplement, 1% GlutaMAX™ supplement, 5 µg/mL transferrin, 10 nM progesterone, 30 µM putrescine, 15 nM sodium selenite, 5 µg/mL insulin, 5 µg/mL heparin, 5 mM lactic acid, 5 ng/mL bFGF, 10 ng/mL platelet-derived growth factor (PDGF), 10 ng/mL neurotrophin 3, and 1% P/S. Well-growing NSC neurospheres were dissociated into single cells using Accutase. The cell suspension was adjusted to a density of 1.2 × 10^6^ cells per 15 mL with the OPC medium and then seeded. After seeding, the cells were cultured in a cell incubator at 37 °C with 5% CO_2_. Half-medium changes were performed every 3–4 days, and passaging was conducted every 7–10 days.

iOPCs at passages 3–5 with good growth status were selected for the induction of differentiation into OLs. First, 24-well plates were coated with PBS containing 100 µg/mL poly-L-ornithine and incubated in a 37 °C cell incubator for at least 4 h. After removing the coating solution, the plates were re-coated with PBS containing 10 µg/mL laminin and incubated overnight at 37 °C. iOPCs were seeded the next day. For conventional in vitro OL differentiation assays, differentiation was induced using an Oligodendrocyte Precursor Cell Differentiation Medium (OPCDM, Cat.#1631, ScienCell, Carlsbad, CA, USA) supplemented with 1% Oligodendrocyte Precursor Cell Differentiation Supplement (OPCDS), 60 ng/mL triiodothyronine (T3), and 1% P/S. For iOPC spontaneous differentiation assays, an iOPC medium without growth factors and OL-promoting differentiation factors was used, which consisted of DMEM/F-12 medium supplemented with the following components: 2% B-27™ supplement, 1% GlutaMAX™ supplement, 5 µg/mL transferrin, 10 nM progesterone, 30 µM putrescine, 15 nM sodium selenite, 5 µg/mL insulin, 5 µg/mL heparin, 5 mM lactic acid, and 1% P/S.

### 2.2. Construction and Infection of Recombinant Lentivirus

The recombinant lentiviral overexpression vector carrying the human *SOX10* gene used in this study was constructed with the assistance of Genechem Co., Ltd. (Shanghai, China). The linearized GV513 vector (Ubi-MCS-CBh-gcGFP-IRES-puromycin, restriction sites BamHI/NheI) was obtained via restriction enzyme digestion. The primer sequences (5′→3′) used to amplify the coding region of the human *SOX10* gene were AGGTCGACTCTAGAGGATCCCGCCACCATGGCGGAGGAGCAGGACCTATC (forward) and ACCGTAAGTTATGTGCTAGCTCACTTGGCGTCGGAGGTGAGGC (reverse).

### 2.3. RT-qPCR

Total RNA was extracted from digested single-cell suspensions using the RNAprep Pure Cell/Bacteria Total RNA Extraction Kit (Cat.# DP430, TIANGEN, Beijing, China), and RNA concentrations were determined using a NanoPhotometer NP80 ultra-micro spectrophotometer (Implen, Munich, Germany). Reverse transcription was performed in a 10 μL reaction containing 400 ng RNA using 5 × PrimeScript RT Master Mix (Cat.# RR036A, Takara, Kusatsu, Japan) on a Chromo4 Multicolor Real-Time PCR Detection System (Bio-Rad, Hercules, CA, USA). PCR primers were synthesized by Beijing Liuhe BGI Co., Ltd. (Beijing, China) with the following sequences (5′→3′): *ACTB* (98 bp): ATCACCATTGGCAATGAGCG (forward, 59.26 °C), TTGAAGGTAGTTTCGTGGAT (reverse, 54.30 °C); *CSPG4* (182 bp): CCTCCTGCTGCAGCTCTACT (forward, 61.33 °C), CTGAGGAGGCGTTCAGAAAC (reverse, 58.57 °C); *OLIG2* (144 bp): GCTCCTCAAATCGCATCCA (forward, 57.91 °C), AAAGGTCATCGGGCTCTG (reverse, 56.63 °C); *PDGFRA* (135 bp): TACACTTGCTATTACAACCACA (forward, 55.40 °C), ATCCTCCACGATGACTAAAT (reverse, 53.52 °C); *SOX10* (196 bp): ATGTCAGATGGGAACCCCGA (forward, 60.62 °C), TGGACATTACCTCGTGGCTG (reverse, 59.75 °C); *ST8SIA1* (90 bp): GGAAATGGTGGGATTCTGAAG (forward, 56.56 °C), TGACAAAGGAGGGAGATTGC (reverse, 57.50 °C). Real-time quantitative PCR was performed in a 25 μL reaction using TB Green Premix Ex Taq™ II (Cat.# RR036A, Takara, Japan) on ice and in the dark. Relative gene expression levels were calculated using the 2^−ΔΔCt^ method.

### 2.4. Immunocytochemistry (ICC)

iOPCs or OLs used for ICC were fixed with 4% paraformaldehyde at room temperature for 15 min. Primary antibodies used in the experiments included the following: A2B5 (mouse, 1:300, MAB312, Millipore, Burlington, MA, USA), GFAP (mouse, 1:300, AB4648, Abcam, Cambridge, UK), Ki67 (rabbit, 1:250, AB16667, Abcam), MBP (mouse, 1:500, AB62631, Abcam), CSPG4 (also known as NG2; rabbit, 1:100, AB5320, Millipore), OLIG2 (rabbit, 1:200, AB9610, Millipore), PDGFR-α (rabbit, 1:300, 5241S, Cell Signaling Technology, Danvers, MA, USA), SOX10 (rabbit, 1:300, AB155279, Abcam), and β-Tubulin-III (mouse, 1:500, AB7751, Abcam). Fluorescent secondary antibodies used included the following: Donkey anti-mouse IgG (H&L) Alexa Fluor^®^ 488 (1:500, AB150105, Abcam), Donkey anti-mouse IgG (H&L) Alexa Fluor^®^ 594 (1:500, AB150108, Abcam), Donkey anti-rabbit IgG (H&L) Alexa Fluor^®^ 488 (1:500, AB150073, Abcam), and Donkey anti-rabbit IgG (H&L) Alexa Fluor^®^ 594 (1:500, AB150076, Abcam). For intracellular antigens, cells were permeabilized with PBS containing 0.3% Triton X-100. A 3% bovine serum albumin solution prepared in sterile PBS was used as the blocking solution and antibody diluent. After blocking for 1 h, cells were incubated with primary antibody diluent overnight at 4 °C. On the next day, after washing three times with PBS, cells were incubated with secondary antibody diluent at room temperature for 2 h. Subsequently, cells were washed three times with PBS and stained with DAPI (1:10, Cat.#28718-90-3, Sigma-Aldrich, Saint Louis, MO, USA) for 10 min. Finally, after three additional washes with PBS, immunofluorescence images were acquired using the DP2-BSW software (version 2.1, Olympus, Tokyo, Japan). All imaging experiments were performed based on three independent cell preparations (*n* = 3 biological replicates). For each biological replicate, three technical replicates were set up, and at least five random fields of view were selected per well. The acquired images were processed using the FIJI software (version 1.54f) to calculate the positive rate of markers and mean fluorescence intensity (MFI). Sholl analysis was performed on MBP^+^ OLs using the Simple Neurite Tracer (SNT) plugin (version 4.2.1) [11].

### 2.5. Transwell Assay

Transwell 24-well cell culture plates (Cat.#3422, Corning Incorporated, Corning, NY, USA) were coated with PBS containing 10 µg/mL fibronectin and 5 µg/mL laminin for 1 h. After coating, control iOPCs and LV-*SOX10*-infected experimental iOPCs were seeded into the upper chambers at two seeding densities (10,000 cells/well and 20,000 cells/well) and incubated in a 37 °C cell incubator with 5% CO_2_ for 18 h to allow spontaneous cell migration in the absence of exogenous chemokines. After 18 h, the old medium was discarded, and cells on the inner surface of the upper chamber were gently removed by wiping with a cotton swab. Then, the cells were fixed with pre-cooled methanol for 15 min, washed with PBS, and stained with DAPI for 10 min. Finally, the cells were rinsed three times in PBS. Fluorescent signals of DAPI-labeled cell nuclei were captured at an emission wavelength range of 450–490 nm under an inverted fluorescence microscope. Each upper chamber was scanned field by field under a 10× objective lens, and the entire well was photographed. Images were processed and counted using the FIJI software.

### 2.6. High-Throughput Drug Screening

Phenotypic screening was performed using HiBit-luciferase technology. First, a pCMV-*SOX10*-HiBit recombinant plasmid was constructed and co-transfected with the LgBit plasmid into HEK293T cells in 15 cm^2^ culture dishes. At 24 h post-transfection, the cells were digested into single-cell suspensions, counted, and seeded into 384-well cell culture screening plates (Cat.#3765, Corning, USA) at a density of 5000 cells/well. Each drug from the FDA compound library (1143 selected compounds from Cat#.L1300, Selleck Chemicals, Houston, TX, USA) was diluted to a final concentration of 5 µM, with 3 biological replicates set. The control group was added with an equal volume of dimethyl sulfoxide (DMSO). After 24 h of drug treatment, detection substrates were added, and luciferase activity signals were detected using a Varioskan LUX multimode microplate reader (VLBL00D0, Thermo Fisher, Waltham, MA, USA). The signal value was used to reflect the SOX10 protein level.

### 2.7. Cell Counting Kit-8 (CCK-8) Assay

Sterile PBS containing 10 µg/mL fibronectin and 5 µg/mL laminin was prepared as a coating solution to coat 5 96-well plates for 1 h. Harvested iOPCs were counted and seeded at a density of 5000 cells/100 µL/well. For the drug stimulation group, an appropriate amount of drug was added to reach the target concentration. The negative control group contained an equal volume of OPC culture medium and cells, while the blank control group contained only an equal volume of OPC culture medium without cells. The plates were then incubated in a cell incubator at 37 °C with 5.0% CO_2_. One plate was removed from the incubator every 24 h, and 10 µL of 10% CCK-8 solution (Cat.#CK04, Dojindo, Kumamoto, Japan) was added to each well. After incubation in the incubator for 4 h, the optical density (OD) value was measured using a multimode microplate reader with a reference wavelength of 650 nm and a test wavelength of 450 nm.

### 2.8. RNA-Seq

Library construction, sequencing, and gene alignment and quantification for RNA-seq were performed with the assistance of Novogene Technology Co., Ltd. (Beijing, China). Qualified libraries were subjected to high-throughput sequencing on an Illumina NovaSeq 6000 sequencing platform (Illumina, San Diego, CA, USA), generating 150 bp paired-end reads. Differential expression analysis was implemented in the R (Version 4.4.2) language environment, and all codes were written and executed in RStudio (Version 2023.06.2 + 561). Differentially expressed gene (DEG) analysis between two comparison groups was performed using the DESeq2 package (Version 1.46.0). Gene Ontology (GO) functional enrichment analysis and Kyoto Encyclopedia of Genes and Genomes (KEGG) pathway enrichment analysis of DEGs between the two comparison groups were conducted using the clusterProfiler package (Version 4.14.6) [12], with the false discovery rate (FDR) controlled via the Benjamini–Hochberg method. An adjusted *p*-value (padj) < 0.05 was used as the threshold for significant enrichment.

### 2.9. Assay for Transposase-Accessible Chromatin with High-Throughput Sequencing (ATAC-Seq)

Library construction, sequencing, and bioinformatics analysis for ATAC-seq were performed with the assistance of Novogene Technology Co., Ltd. High-throughput sequencing was carried out on an Illumina NovaSeq 6000 sequencing platform, producing 150 bp paired-end reads. Peak calling was performed using the MACS2 software (Version 2.2.7.1) with a threshold of q ≤ 0.05 for peak screening. The ChIPseeker package (Version 1.42.1) [13] was loaded in R and RStudio to obtain the nearest gene near each peak and annotate the genomic regions of all screened peaks. The Bedtools software (Version 2.30.0) was used to merge open peaks from different experimental groups, and the mean reads per million mapped reads (RPMs) of each merged peak in each group was calculated. Differential peaks were screened with a threshold of RPM fold change > 2. The ChIPseeker package was used to annotate the genomic regions of differential peaks, followed by GO enrichment analysis and KEGG pathway enrichment analysis using the GOseq package (Version 1.58.0) [14] in R and the KOBAS software (Version 2.0) [15], respectively. For motif analysis, all peak intervals were adjusted to 500 bp sequence fragments centered on the peak summit, and the analysis was completed using the findMotifsGenome.pl program of the Homer software (Version 4.9.1).

### 2.10. Chromatin Immunoprecipitation Sequencing (ChIP-Seq)

Library construction, sequencing, and bioinformatics analysis for ChIP-seq were performed with the assistance of Novogene Technology Co., Ltd. Qualified libraries were subjected to high-throughput sequencing on an Illumina NovaSeq 6000 sequencing platform, generating 150 bp paired-end reads. Peak calling was performed in the MACS2 software with a threshold of q ≤ 0.05 for peak screening. The ChIPseeker package was loaded in R and RStudio to obtain the nearest gene near each peak and annotate the genomic regions. Differential peak analysis was performed using the DiffBind package (Version 3.16.0), and the ChIPseeker package was used to annotate the genomic regions of differential peaks to identify their associated target genes. For the results of peak annotation and differential analysis, GO enrichment analysis and KEGG pathway enrichment analysis were further carried out using the GOseq package in R and the KOBAS software, respectively. Finally, motif analysis of peak sequences was performed using the findMotifsGenome.pl program of the Homer software.

### 2.11. Statistical Analysis

Statistical analysis and graphing were performed using the GraphPad Prism software (version 8.4.0). Quantitative data are presented as the mean ± standard deviation (Mean ± SD), and error bars in all figures represent the standard deviation. All quantitative data were statistically analyzed using independent biological replicates (*n* = 3) as the statistical unit. Normality was assessed using the Shapiro–Wilk test. For normally distributed data, comparisons between two groups were performed using Student’s *t*-test, and comparisons among multiple groups were performed using one-way analysis of variance (ANOVA) followed by Dunnett’s post hoc test. For non-normally distributed data, nonparametric tests were used: the Mann–Whitney U test for two-group comparisons and the Kruskal–Wallis test followed by Dunn’s post hoc test for multiple group comparisons. Statistical significance was defined as *p* < 0.05.

## 3. Results

### 3.1. Differential Gene Expression Profiles Between Endogenous OPCs and In Vitro Cultured iOPCs

Public high-throughput gene expression data associated with OPCs were retrieved from the Gene Expression Omnibus (GEO) database, and the GSE36431 dataset, which includes OPC samples directly isolated from human fetal brain, was chosen as the reference dataset [16]. Among this dataset, three replicates of undifferentiated OPCs (GSM893284, GSM893293, and GSM893304) were chosen to represent endogenous fetal OPCs, and DEG analysis was performed between these samples and RNA-seq data of human iOPCs generated by the in vitro induction of NSCs in our laboratory.

**Figure 1:**
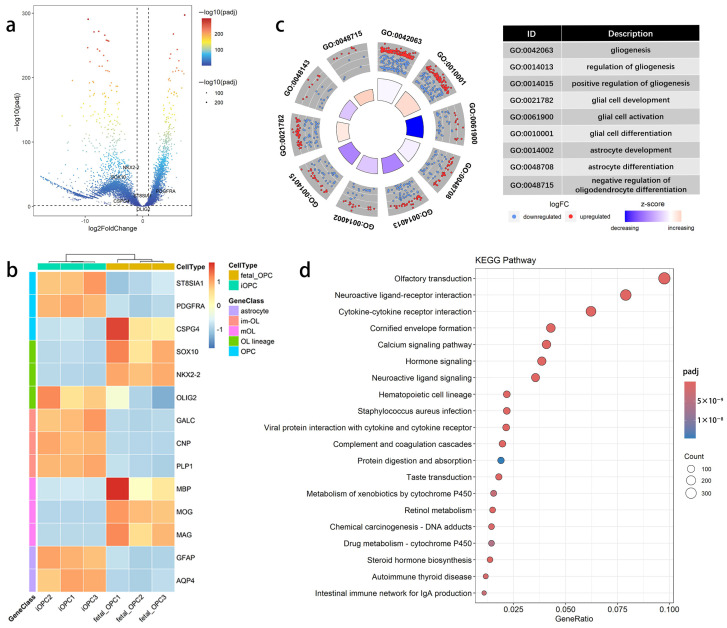
Differential gene expression and functional enrichment analysis between human fetal OPCs and iOPCs. (**a**) Volcano plot of DEGs between human fetal OPCs and iOPCs. The *x*-axis shows the log_2_ fold change in gene expression, and the *y*-axis represents the significance of differential expression; each dot represents one gene. (**b**) Heatmap of glial marker gene expression between human fetal OPCs and iOPCs. The *x*-axis indicates samples, and the *y*-axis indicates genes. (**c**) Circular plot of GO terms related to glial cell function between human fetal OPCs and iOPCs. The outer dots represent DEGs in each GO term; the size of the inner sector corresponds to the number of DEGs, and the color represents the Z-score. (**d**) Bubble plot of KEGG pathway enrichment between human fetal OPCs and iOPCs. The *y*-axis shows pathway names, the *x*-axis shows the ratio of DEGs annotated to each pathway, and the bubble size represents the number of annotated genes. *n* = 3 biological replicates.

With fetal OPCs as the control group and iOPCs as the experimental group, the two datasets were integrated for DEG screening with the criteria of |log_2_ fold change| (|LFC|) ≥ 1 and padj < 0.05, followed by statistical analysis of genes with significantly differential expression levels between OPCs of the two origins. The results showed that, compared with fetal OPCs, 3737 genes (21.3%) were significantly upregulated and 9612 genes (54.7%) were significantly downregulated in iOPCs. These results were visualized as a volcano plot (Figure 1a), with the distribution of key OPC cell markers labeled on the plot simultaneously.

Cell markers serve as specific criteria for cell type identification. We focused on the expression patterns of glial cell marker genes commonly reported in the literature, with the results presented as a heatmap in Figure 1b. For OPC markers, the expression of *OLIG1*, *PDGFRA*, and *ST8SIA1* was markedly upregulated in iOPCs, whereas that of NKX*2-2*, *CSPG4*, and *SOX10* was significantly downregulated; *OLIG2* expression was comparable between the two groups. For OL markers, although iOPCs also exhibited higher expression of several immature OL markers, including *GALC*, *CNP*, and *PLP1*, fetal OPCs showed elevated expression of myelin-related markers for mature OLs, such as *MBP*, *MOG*, and *MAG*. In addition, fetal OPCs displayed lower expression of astrocyte markers (*GFAP* and *AQP4*).

Subsequently, GO functional enrichment analysis and KEGG pathway enrichment analysis were performed on the identified DEGs. Among the 2158 enriched GO terms, nine terms involved in the regulation of glial cell biological processes were further screened out, and Z-scores were calculated for each term to evaluate the global up- and downregulation trends. As shown in Figure 1c, iOPCs exhibited global downregulation in functions, including GO:0014013 regulation of gliogenesis, GO:0014015 positive regulation of gliogenesis, GO:0061900 glial cell activation, and GO:0014002 astrocyte development, whereas global upregulation was observed in GO:0048715 negative regulation of OL differentiation. Among the 80 enriched KEGG pathways, the top 20 most significant pathways were selected to generate a bubble plot (Figure 1d). Obvious differences in activation and expression were observed between the two types of OPCs in several key signaling pathways, including the hsa04151 PI3K-Akt signaling pathway, hsa04063 JAK-STAT signaling pathway, and hsa04024 cAMP signaling pathway.

### 3.2. Insufficient Activation of Core Transcription Factor SOX10 in iOPCs

**Figure 2:**
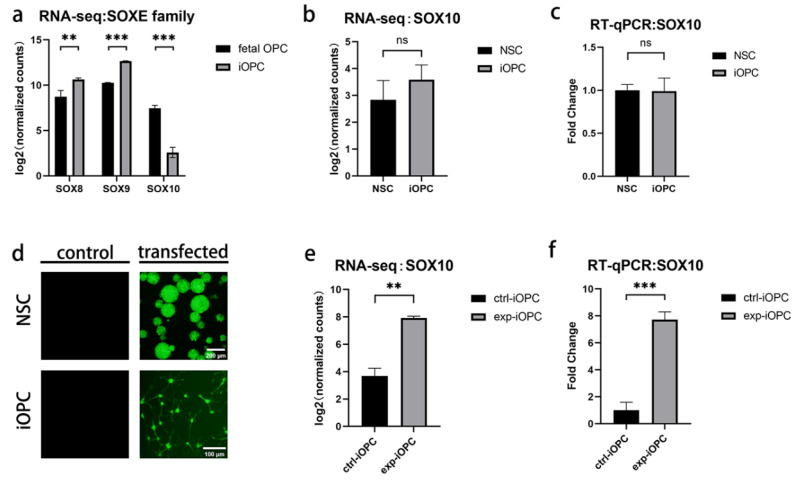
Expression of *SOX10* in different OPCs and NSCs. (**a**) Expression differences of *SOXE* family genes in human fetal OPCs and iOPCs; the *y*-axis shows log-transformed expression levels. (**b**) *SOX10* expression in iOPCs and NSCs detected by RNA-seq; the *y*-axis shows log-transformed expression levels. (**c**) *SOX10* expression in iOPCs and NSCs was validated via RT-qPCR; normalized to ACTB and relative to NSCs, the *y*-axis shows fold change in expression. (**d**) GFP expression in NSCs infected with *SOX10*-GFP lentivirus and their derived iOPCs (experimental group) after puromycin selection; scale bars: 200 µm for NSCs; 100 µm for iOPCs. (**e**) *SOX10* expression in control and experimental iOPCs detected by RNA-seq; the *y*-axis shows log-transformed expression levels. (**f**) *SOX10* expression in control and experimental iOPCs validated via RT-qPCR, normalized to ACTB and relative to control iOPCs; the *y*-axis shows fold change in expression. *n* = 3 biological replicates. ns = not significant; ** *p* < 0.01, and *** *p* < 0.001.

Transcription factors involved in the development and differentiation of OPCs were our major focus. In the present study, *OLIG2* was not a differentially expressed gene between groups compared with fetal OPCs; the expression of the *SOXE* family members *SOX8* and *SOX9* was significantly upregulated in iOPCs, whereas *SOX10* expression was significantly downregulated (Figure 2a). We also performed RNA sequencing on NSCs, the source cells of iOPCs. Analyses of differentially expressed genes between iOPCs and NSCs revealed that there was no significant difference in *SOX10* expression during the induction from NSCs to iOPCs (Figure 2b). Furthermore, RT-qPCR assays verified the expression of *SOX10* between iOPCs and NSCs, showing that *SOX10* was not significantly activated (Figure 2c).

To investigate the function of SOX10 during iOPC induction from NSCs, we constructed a lentiviral vector carrying the *SOX10* gene, GFP, and a puromycin resistance gene, which was successfully used to infect NSCs. The transgenic NSCs were then continuously passaged in the NSC medium containing puromycin for screening and purification, and then induced into experimental iOPCs with early *SOX10* overexpression using the OPC induction medium. The transgenic NSCs and their induced experimental iOPCs were observed under a fluorescence microscope. Green fluorescence produced by GFP expression was visible in NSCs in the form of cell spheres, and it was mainly concentrated in the nucleus of experimental iOPCs, while no fluorescence was observed in non-transgenic NSCs and their induced control iOPCs (Figure 2d). Subsequently, we verified the changes in *SOX10* expression in experimental iOPCs. RNA-seq results showed that compared with control iOPCs, the expression of *SOX10* was significantly increased in experimental iOPCs (Figure 2e). The RT-qPCR results also demonstrated that the expression level of *SOX10* mRNA in experimental iOPCs was 7.7-fold higher than that in control iOPCs (Figure 2f).

### 3.3. Effects of Early SOX10 Overexpression on the Phenotypic Characteristics of iOPCs

**Figure 3:**
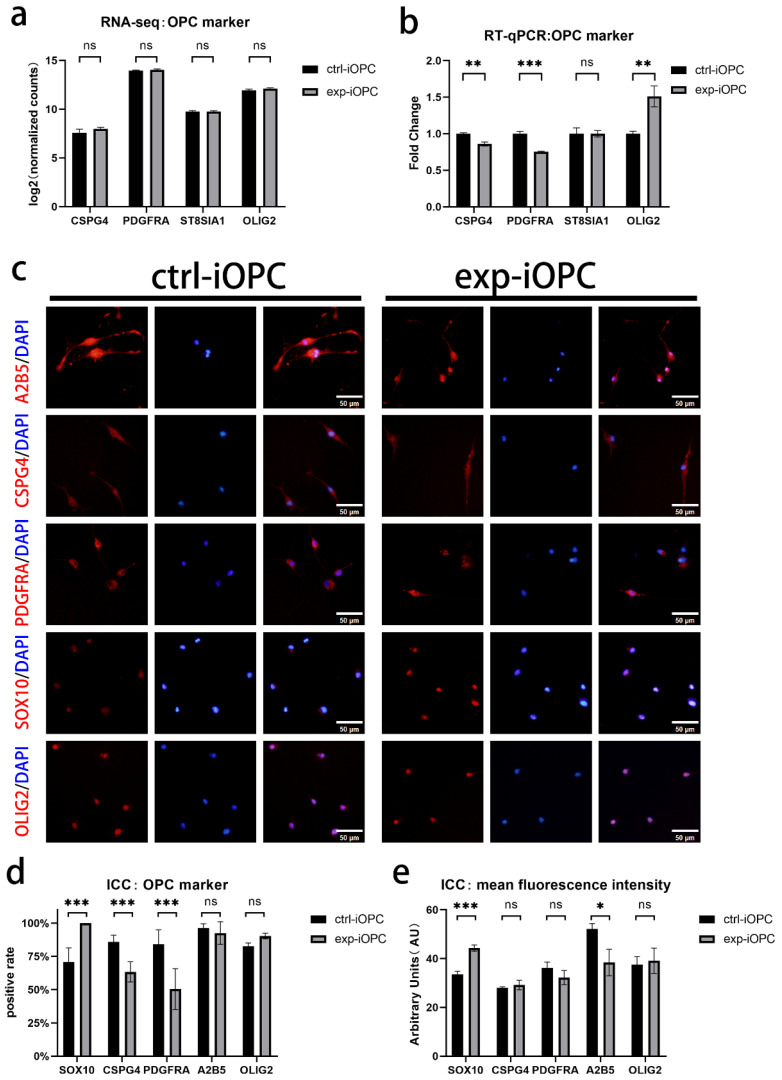
Expression profiling of signature OPC markers in experimental and control iOPCs at transcriptional and cellular levels. (**a**) RNA-seq analysis of OPC marker gene expression in control and experimental iOPCs; the *y*-axis shows log_2_-transformed normalized counts. (**b**) RT-qPCR validation of signature marker gene expression in control and experimental iOPCs; the *y*-axis shows fold change relative to control iOPCs, normalized to ACTB. (**c**) Representative ICC images of OPC markers (red), DAPI-stained nuclei (blue), and merged signals showing pink overlap in control and experimental iOPCs; scale bar = 50 µm. (**d**) ICC analysis of positive cell rate for each OPC marker; the *y*-axis shows the percentage of marker-positive cells (%). (**e**) MFI of each OPC marker; the *y*-axis shows fluorescence intensity in arbitrary units (AU). *n* = 3 biological replicates. ns = not significant; * *p* < 0.05, ** *p* < 0.01, and *** *p* < 0.001.

We also examined and validated the expression changes of other key OPC markers in experimental and control iOPCs. At the transcriptional level, DEG analysis of RNA-seq data revealed no significant differences in the expression of OPC signature marker-associated genes, including *CSPG4* (*NG2*), *PDGFRA*, *ST8SIA1* (A2B5), and *OLIG2*, between experimental and control iOPCs (Figure 3a). RT-qPCR assays were further performed to verify the mRNA expression of these OPC markers, and the results showed a mild decrease in the mRNA levels of *CSPG4* and *PDGFRA*, a slight increase in *OLIG2* expression, and no statistically significant difference in *ST8SIA1* expression in experimental iOPCs relative to controls (Figure 3b).

At the cellular level, we quantitatively assessed whether the expression of OPC markers was markedly altered in experimental iOPCs by analyzing two parameters: the positive cell rate and the global expression level of each marker. ICC staining results demonstrated that both experimental and control iOPCs expressed the OPC-specific markers SOX10, CSPG4, PDGFRA, A2B5, and OLIG2 (Figure 3c). However, for the positive cell rate, experimental iOPCs exhibited a significant increase in the proportion of SOX10-positive cells, a decrease in the positive rates of CSPG4 and PDGFRA, and no notable differences in the positive rates of A2B5 and OLIG2 compared with control iOPCs (Figure 3d). Fluorescent images of cellular markers in each group were further processed using the FIJI software to calculate the MFI, which allowed semi-quantitative analysis of the global expression level of each marker in positive cells. The results showed that SOX10-positive experimental iOPCs had a higher SOX10 expression level, A2B5-positive experimental iOPCs displayed a reduced A2B5 expression level, and no intergroup differences were observed in the expression levels of PDGFRA, CSPG4, and OLIG2 (Figure 3e).

**Figure 4:**
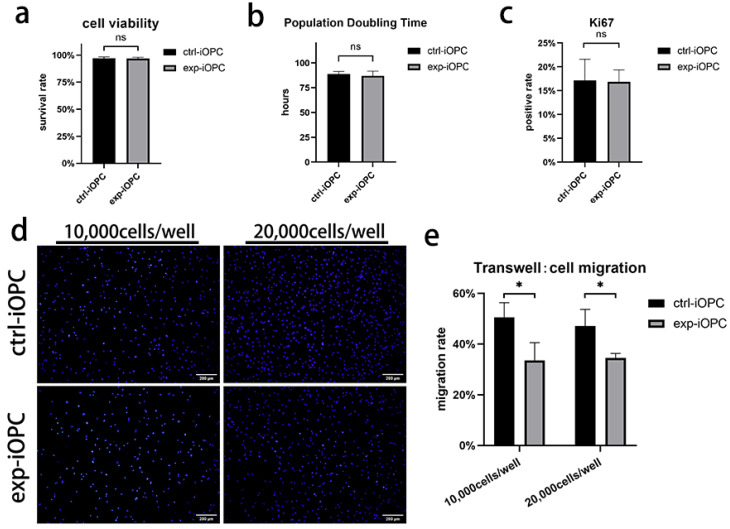
Effects of early *SOX10* overexpression on iOPC survival, proliferation, and migration. (**a**) Cell viability of control and experimental iOPCs during continuous subculture. (**b**) Population doubling time of control and experimental iOPCs. (**c**) Ki67-positive rate of control and experimental iOPCs. (**d**) Representative images from the Transwell migration assay. Migrated cell nuclei were stained with DAPI (blue); scale bar = 200 µm. (**e**) Quantitative analysis of migration rate at two seeding densities (10,000 cells/well and 20,000 cells/well). *n* = 3 biological replicates. ns = not significant; * *p* < 0.05.

To evaluate the effects of early *SOX10* overexpression on the survival and proliferative capacity of iOPCs, continuous subculture was performed on control and experimental iOPCs. During subculture, cell viability was compared between the two groups via trypan blue staining and cell counting, and the results showed comparable viability in control and experimental iOPCs (Figure 4a). The proliferative capacity of cells was assessed by calculating the population doubling time (PDT) of cultured cells. When seeded at a standard density (2 × 10^4^ cells/cm^2^), experimental iOPCs exhibited no significant difference in proliferation efficiency relative to control iOPCs (Figure 4b). Ki67 is a proliferation-associated antigen expressed in the S, G1, G2, and M phases of the cell cycle, serving as a robust marker for reflecting cellular proliferative status. Ki67 staining of control and experimental iOPCs revealed no notable difference in the proportion of proliferating cells between the two groups at the same post-seeding time points (Figure 4c). As OPCs are migratory stem cells, Transwell assays were conducted to evaluate the migratory capacity of control and experimental iOPCs. Considering the potential effect of seeding density on migration efficiency, two seeding densities were set for each group (10,000 cells/well and 20,000 cells/well in 24-well plates). The results demonstrated that seeding density had no obvious impact on the migration rate of iOPCs in either group; however, experimental iOPCs displayed a significantly lower migration rate than control iOPCs at both seeding densities (Figure 4d,e).

### 3.4. Early SOX10 Overexpression Promotes the Differentiation of iOPCs into Mature OLs

**Figure 5:**
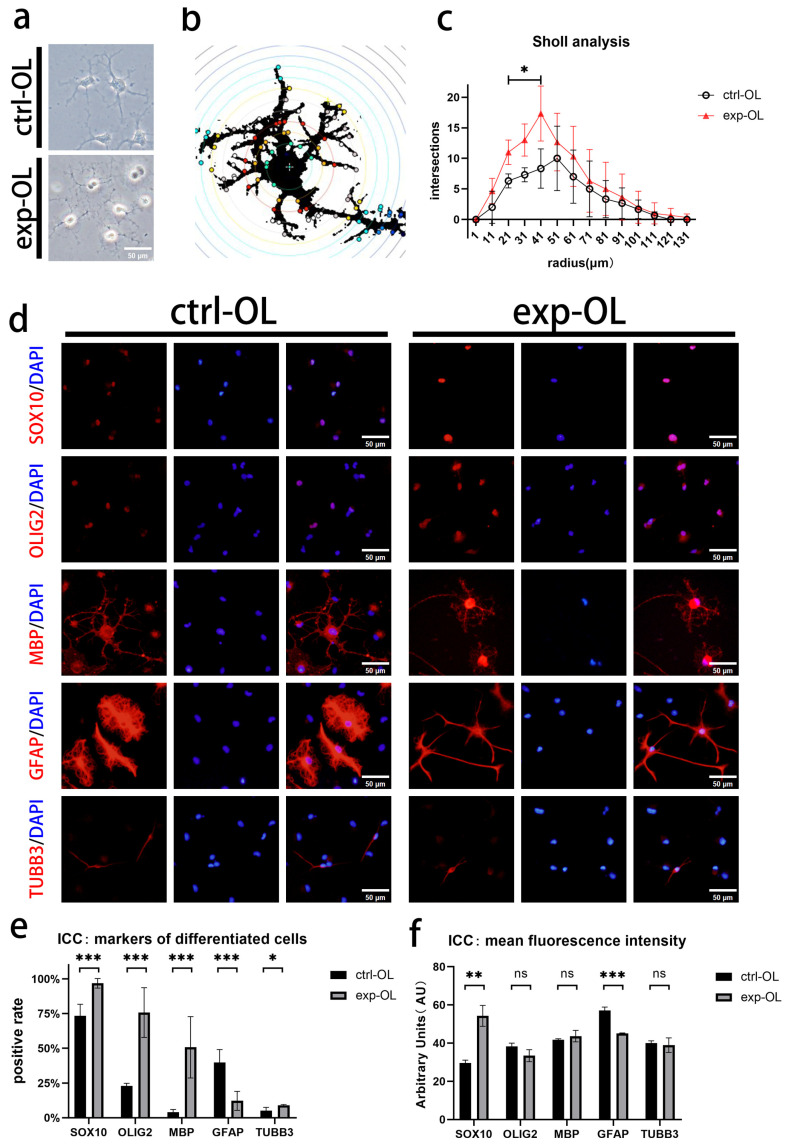
Early *SOX10* overexpression promotes iOPC differentiation into mature OLs. (**a**) Morphology of OLs differentiated from control and experimental iOPCs (day 14); scale bar = 50 µm. (**b**) Schematic of Sholl analysis for MBP^+^ OLs: concentric circles centered on the nucleus, with intersections reflecting branching complexity. (**c**) Quantitative Sholl analysis plot; the *x*-axis shows distance from the soma (radius), and the *y*-axis shows the number of intersections. (**d**) Representative ICC images of differentiated cell lineage markers (OL, astrocyte, and neuronal markers) in cells spontaneously differentiated from control and experimental iOPCs. Markers are shown in red, nuclei are counterstained with DAPI (blue), and merged signals appear pink due to overlap; scale bar = 50 µm. (**e**) Positive cell rate of each lineage marker in differentiated cells; the *y*-axis shows percentage of marker-positive cells (%). (**f**) MFI of each lineage marker in differentiated cells; the *y*-axis shows fluorescence intensity in AU. *n* = 3 biological replicates. ns = not significant; * *p* < 0.05, ** *p* < 0.01, and *** *p* < 0.001.

To preliminarily verify whether *SOX10*-overexpressing iOPCs retain their OL differentiation capacity, we induced control and experimental iOPCs to differentiate into OLs in vitro using OPCDM supplemented with pro-differentiation factors. Under microscopy, mature OLs with typical branched morphology appeared in both groups within 2 weeks. Notably, OLs derived from experimental iOPCs formed more abundant secondary branches and began to develop membrane sheet-like structures at the same time point (Figure 5a).

To further explore the effect of early *SOX10* overexpression on the differentiation potential of iOPCs under a more in vivo-like microenvironment, the two groups of cells were subjected to spontaneous differentiation for 14 days in OL spontaneous differentiation medium without differentiation-promoting factors, followed by immunocytochemical staining. Representative staining results are shown in Figure 5d. Compared with the control group, differentiated cells from the experimental iOPCs exhibited higher positive rates of SOX10, OLIG2, MBP, and the neuronal marker TUBB3, whereas the positive rate of GFAP was significantly decreased (Figure 5e). Quantification of the MFI of each marker revealed significantly elevated SOX10 protein expression and reduced GFAP protein expression in differentiated cells of the experimental group, with no statistically significant differences in the expression of OLIG2, MBP, and TUBB3 between the two groups (Figure 5f). OL maturation is positively correlated with morphological complexity, which can therefore be used to evaluate the maturation status of OLs [17]. Sholl analysis was performed on immunofluorescent images of MBP^+^ OLs from both groups. The results showed that the number of branches was significantly higher in the experimental group at the same radial distance (Figure 5b,c), indicating that OLs differentiated from experimental iOPCs display a more complex branching phenotype and a more mature morphological profile.

### 3.5. SOX10 Target Genes Exhibit Extensive Chromatin Accessibility

**Figure 6:**
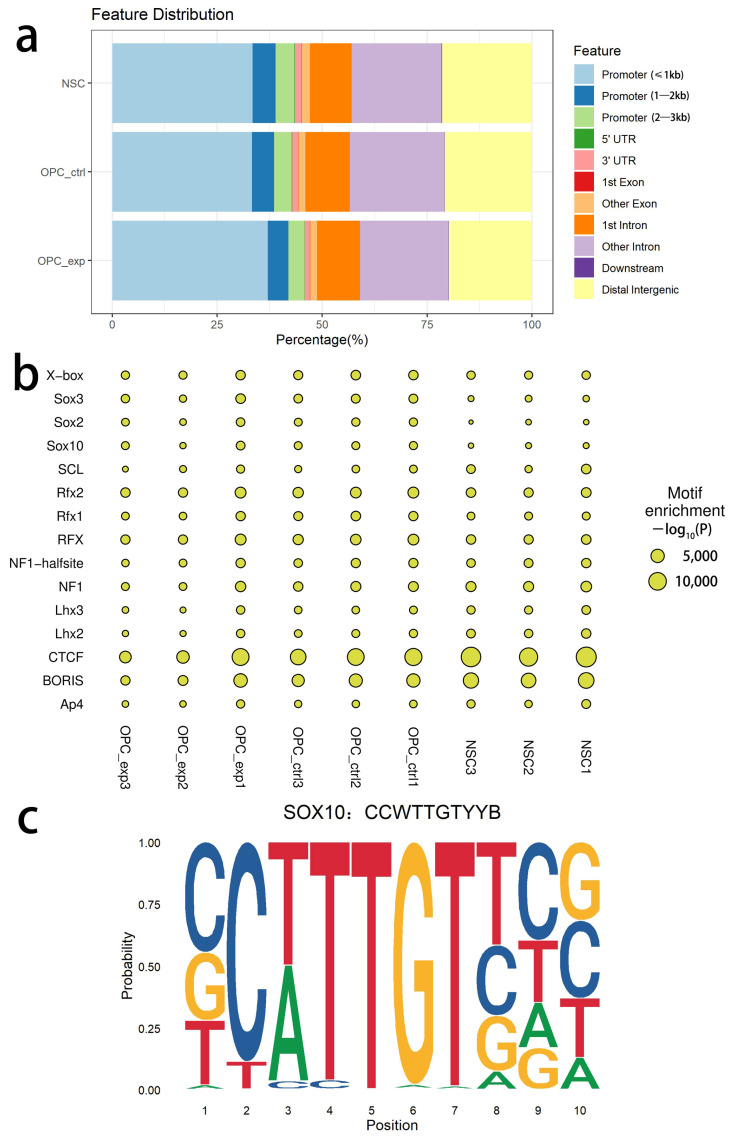
ATAC-seq analysis reveals chromatin accessibility and transcription factor motif enrichment in NSCs and iOPCs. (**a**) Genomic distribution of ATAC-seq peaks in NSCs, control iOPCs, and experimental iOPCs. The *x*-axis shows the percentage of peaks in each category, and different colors represent distinct genomic features. (**b**) Transcription factor motif enrichment plot across biological replicates of NSCs, control iOPCs, and experimental iOPCs. Cell samples are shown on the *x*-axis, transcription factor motifs are shown on the *y*-axis, and the size of each circle indicates motif enrichment level. (**c**) Sequence logo of the SOX10 motif identified by motif enrichment analysis, with the consensus sequence CCWTTGTYYB. Nucleotide positions are shown on the *x*-axis, and the probability of each nucleotide at each position is shown on the *y*-axis. *n* = 3 biological replicates.

Although functional differences existed between experimental and control iOPCs, the actual functional effects of ectopically expressed SOX10 were closely associated with chromatin accessibility. Therefore, we performed ATAC-seq analysis on NSCs, control iOPCs, and experimental iOPCs. For the ATAC-seq data, genomic regions with significantly enriched reads aligned with the reference genome were defined as peaks and identified by statistical analysis. As shown in Figure 6a, peaks in NSCs, control iOPCs, and experimental iOPCs were mainly distributed in promoter regions. Specifically, the promoter distribution in NSCs was 33.4% at ≤1 kb, 5.5% at 1–2 kb, and 4.5% at 2–3 kb relative to the TSS; in control iOPCs, it was 33.3% at ≤1 kb, 5.2% at 1–2 kb, and 4.2% at 2–3 kb; in experimental iOPCs, it was 37.0% at ≤1 kb, 4.9% at 1–2 kb, and 3.8% at 2–3 kb.

Transcription factors and other regulators control gene transcription by recognizing and binding to specific conserved DNA sequences (known as motifs), and their binding sites are termed transcription factor binding sites (TFBSs). The Homer software was used to identify the enriched motif sequence features in peak regions, thereby recognizing transcription factors that may bind to open chromatin regions. As shown in Figure 6b,c, motifs corresponding to transcription factors, including SOX2, SOX3, SOX10, RFX, and CTCF, exhibited the highest enrichment in all cell samples. These results suggest that SOX10 has extensive potential binding targets in both NSCs and OPCs and indicate that these transcription factors may function throughout the induction of NSCs into iOPCs.

### 3.6. Significant Remodeling of SOX10 Binding Pattern Under Minor Transcriptional Level Changes

**Figure 7:**
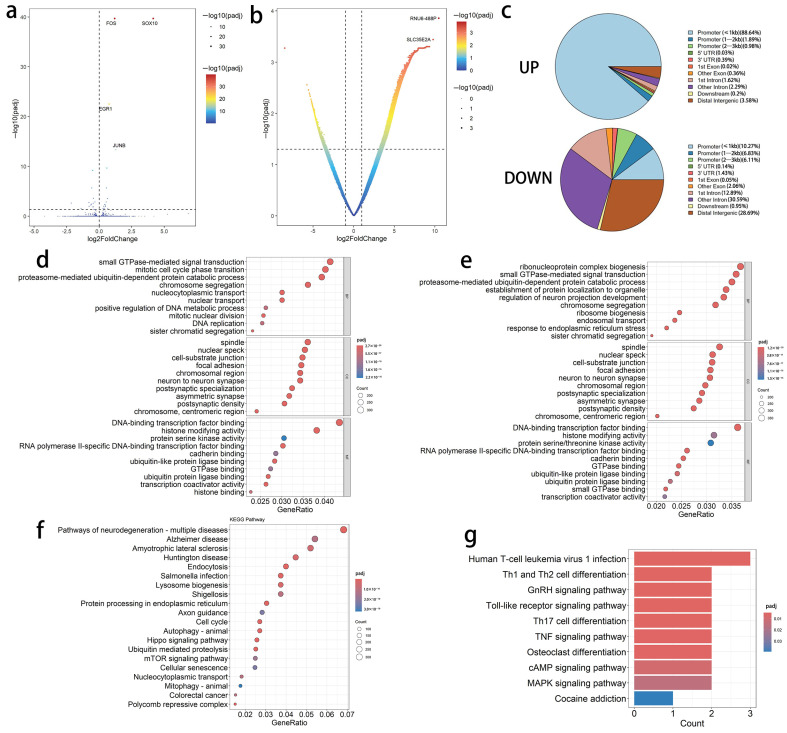
Multi-omics analysis reveals the regulatory landscape of DEGs and binding regions in experimental iOPCs. (**a**) Volcano plot of DEGs from RNA-seq between experimental and control iOPCs. (**b**) Volcano plot of differential chromatin accessibility peaks between experimental and control iOPCs. (**c**) Genomic distribution of up- and downregulated DBRs. (**d**) GO enrichment of genes linked to upregulated DBRs. (**e**) GO enrichment of genes overlapping between ATAC-seq and ChIP-seq peaks in experimental iOPCs. (**f**) KEGG pathway enrichment of genes overlapping between ATAC-seq and ChIP-seq peaks in experimental iOPCs. (**g**) KEGG enrichment results of downstream target genes directly upregulated by SOX10 binding.

To investigate the effect of early *SOX10* overexpression on the transcription of other genes in iOPCs, DEG analysis via RNA-seq was performed between experimental iOPCs and control iOPCs. DEGs were screened with the criteria of |LFC| > 0 and padj < 0.05. The results revealed that, compared with the control group, 15 genes were significantly upregulated and another 15 were markedly downregulated in the experimental iOPCs (Figure 7a).

Given that transcription factor binding and regulation represent a pivotal step in transcriptional initiation, ChIP-seq with a SOX10-specific antibody was subsequently conducted to further explore the potential intergroup differences in regulatory patterns. Differential ChIP-seq peaks between the two groups reflect open genomic regions with distinct binding affinities to SOX10, defined as differential binding regions (DBRs). For the identification of intergroup differences in SOX10 binding loci, DBRs were filtered using the stringent criteria of |LFC| ≥ 1 and padj < 0.05. As shown in Figure 7b, 9788 DBRs were significantly upregulated, and 4322 DBRs were remarkably downregulated in the experimental group relative to the control group. Additionally, the genomic distribution of these differential peaks across functional regions exhibited significant intergroup disparities (Figure 7c); in particular, the upregulated differential peaks in the experimental iOPCs were predominantly localized to promoter regions, whereas the downregulated differential peaks were largely distributed in intronic and intergenic regions.

GO functional enrichment analysis was performed on the genes associated with these DBRs. A total of 2185 GO terms were enriched in the upregulated peaks (Figure 7d), among which 14 GO terms related to the regulation of glial cell biological processes were identified in the significantly enriched upregulated BP terms, including “GO:0021781 glial cell fate commitment” and “GO:0048713 regulation of oligodendrocyte differentiation”. In contrast, only 264 GO terms were enriched in the genes associated with downregulated peaks, and no GO terms related to the regulation of glial cell biological processes were detected.

Open chromatin is a prerequisite for the binding of most transcription factors. Therefore, the overlapping regions between chromatin open regions identified by ATAC-seq and protein-binding sites identified by ChIP-seq typically possess greater potential for actual regulatory activity. The GenomicRanges package in R was used to calculate the overlapping regions between open peaks from ATAC-seq and enriched peaks from ChIP-seq in two groups of iOPCs. The genes associated with these overlapping regions are more likely to be downstream target genes that the transcription factor SOX10 binds to and exerts its regulatory functions on. In the control iOPCs, there were 1049 overlapping regions between ATAC-seq-enriched peaks and ChIP-seq-enriched peaks with an average length of 210 bp, which were annotated to 739 non-redundant associated genes. GO and KEGG functional analyses were performed on these 739 common genes. The results showed that only nine synapse-related cellular component (CC) terms were enriched, while no cell functions closely related to OLs were enriched. In addition, no significantly enriched pathways were identified in the KEGG enrichment analysis. In the experimental iOPCs, there were 21,030 overlapping regions between ATAC-seq enriched peaks and ChIP-seq enriched peaks, with an average length of 593 bp, which were annotated to 12,581 non-redundant associated genes. Functional enrichment analysis revealed that the SOX10 target genes in the experimental iOPCs were enriched in 2426 GO terms (Figure 7e), including biological processes related to OL function, such as “GO:0048505 regulation of cell differentiation timing” and “GO:0048713 regulation of oligodendrocyte differentiation”. Meanwhile, a total of 199 KEGG pathways were enriched in the KEGG pathway enrichment analysis (Figure 7f), including “hsa04150 mTOR signaling pathway”, “hsa04310 Wnt signaling pathway”, “hsa04012 ErbB signaling pathway”, and “hsa04330 Notch signaling pathway”.

With a significance threshold set at q < 0.05, motif analysis of DBRs revealed that 400 distinct motifs were significantly enriched in the upregulated DBRs of iOPCs in the experimental group. Notably, the conserved motif directly recognized by SOX10 was among these enriched motifs, accounting for 22.01% of the total target sequences within the upregulated DBRs. The other most highly enriched motifs in the upregulated DBRs included YY1 (CAAGATGGCGGC), ELK4 (NRYTTCCGGY), ELK1 (HACTTCCGGY), ETS (AACCGGAAGT), and FLI1 (NRYTTCCGGH) (Table 1). In contrast, only nine motifs were enriched in the downregulated DBRs of experimental iOPCs, and the SOX10-specific motif was not detected in these regions. The top enriched motifs in the downregulated DBRs comprised SREBP1A, SREBP2, HNF1B, KLF10, and the FOXA1:AR heterodimer.

We performed integrated analysis of ChIP-seq and RNA-seq data to establish the correlation between SOX10-occupied TFBS and the dynamic expression of their target genes, thereby identifying candidate target genes with differential SOX10 binding accompanied by altered expression. We intersected upregulated DBR-annotated genes in the promoter regions of iOPCs in the experimental group with intergroup DEGs identified by RNA-seq. The expression of six genes (FOS, JUN, EGR1, RHOB, ANGPTL2, and SNRPG) was positively correlated with enhanced SOX10 binding in their promoter regions; conversely, the expression of another six genes (CALR, MANF, HSPA5, HSP90B1, LHX2, and LAMA1) was negatively correlated with increased SOX10 promoter binding. However, intersection analysis of downregulated DBR-annotated genes in the promoter regions of iOPCs in the experimental group and DEGs yielded no overlapping genes.

We also conducted functional enrichment analysis on these downstream target genes directly bound and regulated by SOX10 to characterize the functional pathways directly controlled by SOX10. KEGG pathway enrichment analyses of directly upregulated target genes revealed 42 pathways (Figure 7g), including multiple signaling pathways, such as the “hsa04010 MAPK signaling pathway” and “hsa04024 cAMP signaling pathway”, for which their enriched genes were closely related to FOS and JUN. Only four minor pathways were identified in the KEGG pathway enrichment analysis of downregulated target genes.

We further investigated whether intergroup changes in chromatin accessibility in iOPCs directly contribute to the altered binding sites of the transcription factor SOX10. The GenomicRanges package in R was used to calculate the overlapping regions between upregulated differentially accessible regions (DARs) from ATAC-seq and upregulated DBRs from ChIP-seq in iOPCs of the experimental group compared with the control group. Only four overlapping peaks were identified, with an average length of 223 bp, and these peaks were not associated with relevant genes. These results indicate that increased chromatin accessibility is not directly correlated with enhanced transcription factor binding, and elevated chromatin accessibility at target gene loci is not the direct cause of SOX10 binding to promoter regions.

### 3.7. Small-Molecule Drugs Promote SOX10 Gene Expression in iOPCs

**Figure 8:**
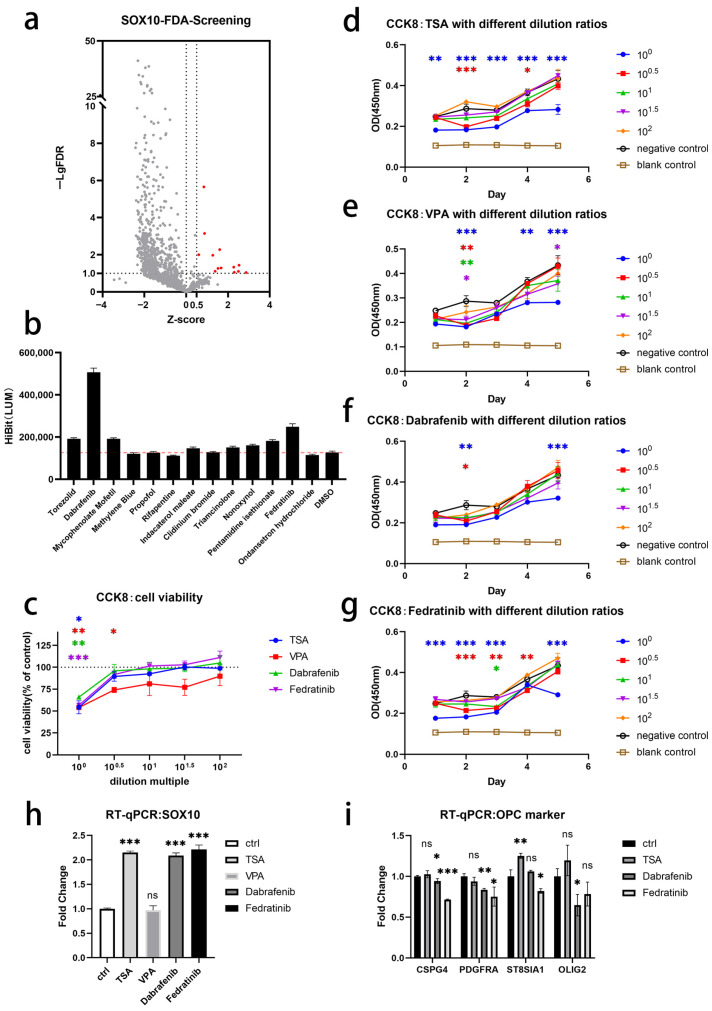
Screening and validation of small-molecule drugs promoting *SOX10* expression in iOPCs. (**a**) High-throughput screening of *SOX10*-upregulating small-molecule drugs, with *X*-axis representing Z-score and *Y*-axis representing -Lg FDR; red dots indicate positive drugs (Z-score > 0.5, -Lg FDR > 1.0). (**b**) HiBit signal in drug-treated iOPCs, with DMSO treatment as the negative control. (**c**) Dose–response curves of 72 h cell viability measured by CCK-8 assay. The *x*-axis shows the drug dilution factor, and the *y*-axis shows relative cell viability (%). Cell viability was calculated relative to the negative control group, which was set to 100% (dashed line). Colored asterisks denote statistical significance between the color-matched experimental group and the negative control at each concentration. (**d**–**g**) Time-course curves of cell viability under different drug concentrations. The *x*-axis shows culture time, and the *y*-axis shows OD450 values representing the relative cell number. Colored asterisks denote statistical significance between the color-matched experimental group and the negative control at the indicated time points. (**h**) *SOX10* expression after treatment with four candidate drugs (TSA, VPA, Dabrafenib, and Fedratinib) detected via RT-qPCR. Data were normalized to ACTB and compared with control iOPCs. (**i**) Effects of three effective drugs (TSA, Dabrafenib, and Fedratinib) on the expression of other OPC marker genes detected via RT-qPCR. *n* = 3 biological replicates. ns = Not significant; * *p* < 0.05, ** *p* < 0.01, and *** *p* < 0.001. Non-significant values are not displayed when group comparisons are numerous for better visualization.

Although we demonstrated the critical role of SOX10 in the induction of iOPCs from NSCs via transgenic experiments, the application of transgenic technology still faces numerous obstacles that need to be overcome, with limited clinical applicability. Therefore, exploring appropriate small-molecule drug interventions to enhance *SOX10* gene expression represents a safer alternative approach. Using “SOX10” as the keyword to search the PubChem database, two histone deacetylase inhibitors (HDACis), TSA and VPA, which can increase *SOX10* mRNA expression, were selected as candidates [18,19]. In addition, a total of 1143 drugs were used for high-throughput drug screening, with HEK293T cells as the screening model and DMSO added to the control group for comparison. Using Z-score > 0.5 and -Lg FDR > 1.0 as the criteria for positive drugs, the results showed that only 13 drugs could upregulate *SOX10* gene expression in HEK293T cells (Figure 8a). Among these 13 drugs (Figure 8b), the two most effective drugs—namely, Dabrafenib (CAS: 1195765-45-7) and Fedratinib (CAS: 936091-26-8)—were selected for further experiments.

Four drugs (TSA, VPA, Dabrafenib, and Fedratinib) were chosen as candidate additives for subsequent experiments to enhance *SOX10* gene expression in iOPCs at the early stage of induction from NSCs. Since OPCs are stem cells sensitive to the living environment, it is necessary to first explore the appropriate drug concentrations suitable for OPC survival to reduce the impact of drug toxicity on cell viability. Referring to the safe drug concentrations of these drugs in other cell experiments [18,20,21], the initial maximum concentrations for iOPC induction culture were preset as follows: TSA (0.1 µM), VPA (3000 µM), Dabrafenib (10 µM), and Fedratinib (1 µM). Then, five concentration gradients were prepared via geometric dilution at a multiple of 10, and the CCK8 kit was used to quantitatively evaluate the changes in cell viability when cultured with different concentrations of drugs. iOPCs induced and cultured in the regular OPC medium served as the negative control group, and the regular OPC medium without cells served as the blank control group. iOPCs induced and cultured in the OPC medium supplemented with different concentrations of each small-molecule drug served as the experimental groups. The dose–response curves measured 72 h after cell seeding showed that all four drugs exhibited significant inhibitory effects on cell viability at the initial maximum concentration, and cell viability improved significantly after 10-fold dilution (Figure 8c). The time-course curves of the four drugs showed that when added at low concentrations, the drug-treated groups maintained comparable cell proliferation capacity to the control iOPCs (Figure 8d–g).

Based on the CCK8 assay results, TSA (0.01 µM), VPA (300 µM), Dabrafenib (1 µM), and Fedratinib (0.1 µM) were selected as the drug concentrations for subsequent experiments. The actual effects of the four initially screened drugs on iOPCs were verified via RT-qPCR experiments. With iOPCs induced and cultured in the regular OPC medium as the control group, the results showed that the three drug-treated groups (TSA, Dabrafenib, and Fedratinib) significantly promoted *SOX10* mRNA expression, while the VPA group showed no obvious change (Figure 8h). For the three drug-treated groups (TSA, Dabrafenib, and Fedratinib) that successfully promoted *SOX10* gene expression, their effects on other important OPC surface markers were further identified. The RT-qPCR results showed that compared with the control group, TSA addition slightly promoted ST8SIA1 gene expression, Dabrafenib addition slightly inhibited the expression of three genes (CSPG4, PDGFRA, and OLIG2), and Fedratinib addition slightly inhibited the expression of three genes (CSPG4, PDGFRA, and ST8SIA1) (Figure 8i). Overall, the effects of the three drugs on these OPC surface markers were not significant.

**Figure 9:**
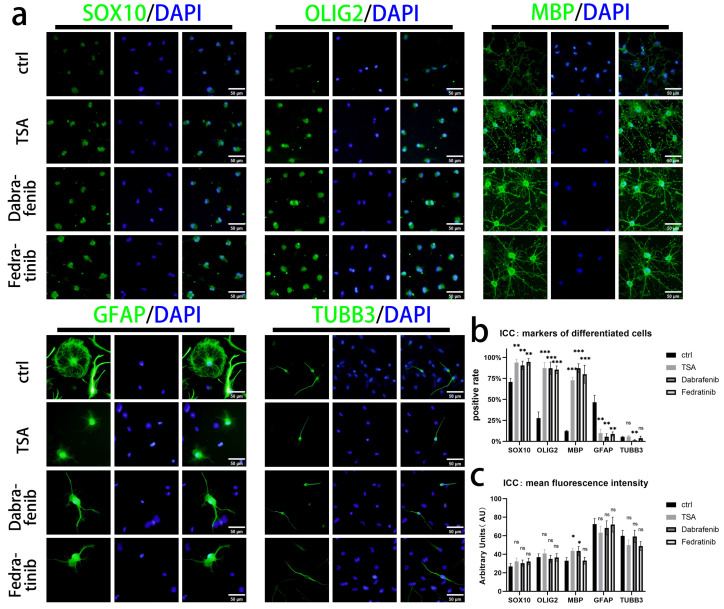
Differences in immunological expression of lineage markers in differentiated iOPCs treated with small-molecule drugs. (**a**) Representative ICC images of each marker in cells spontaneously differentiated from control iOPCs and iOPCs treated with three small-molecule drugs (TSA, Dabrafenib, and Fedratinib). Markers are labeled in green, and nuclei are counterstained with DAPI (blue); scale bar = 50 µm. (**b**) Comparison of the positive rates of lineage markers in differentiated cells among groups. (**c**) Comparison of the MFI of markers in differentiated cells among groups; the y-axis shows fluorescence intensity in AU. *n* = 3 biological replicates. ns = not significant; * *p* < 0.05, ** *p* < 0.01, and *** *p* < 0.001.

We finally examined the differentiation potential of iOPCs treated with the three drugs TSA, Dabrafenib, and Fedratinib. Control and drug-treated iOPCs were allowed to self-differentiate for 14 days in a differentiation medium without pro-differentiation factors. After cell fixation, ICC was performed for identification, and the results are shown in Figure 9a. Compared with the control group, the differentiated iOPCs in all three drug-treated groups exhibited significantly higher positive rates of SOX10, OLIG2, and MBP and a markedly lower positive rate of GFAP. The Dabrafenib group also showed a slight decrease in the positive rate of TUBB3 (Figure 9b). Analyses of the MFI of each marker revealed that the mean expression level of MBP in MBP^+^ cells was increased in the TSA and Dabrafenib groups, while no significant intergroup differences were observed for the other markers (Figure 9c).

## 4. Discussion

In 1981, Stallcup et al. developed an antibody (named NG2) targeting chondroitin sulfate proteoglycan (CSPG), through which they identified the fourth type of glial cell, namely, NG2 glia, also known as OPCs [22]. A large number of subsequent studies have further revealed a series of characteristics of OPCs, including their morphology, molecular phenotype, gene expression profile, function, and differentiation potential. However, despite being collectively referred to as OPCs, cells derived from different sources, at different developmental stages, or with different distribution sites exhibit inconsistencies in morphology and function, a phenomenon defined as OPC heterogeneity [9]. When inducing in vitro myelination of mouse OPCs, Bechler et al. found that the intrinsic length of myelin formed by spinal cord-derived OPCs was greater than that by cortex-derived OPCs, while the number of myelinated segments was roughly comparable, indicating that the heterogeneity of OPC origin had predetermined the intrinsic differentiation potential of OPCs [23].

Differences in gene transcriptional regulation constitute the fundamental cause of OPC heterogeneity. By comparing the gene expression profiles of endogenous fetal OPCs and in vitro-generated iOPCs, we identified substantial global transcriptional differences between the two cell types. Despite unavoidable technical bias and biological heterogeneity across datasets, glial marker genes displayed specific rather than uniform expression changes, suggesting that these differences genuinely reflect distinct cellular characteristics of fetal OPCs and iOPCs. In terms of differentiation-related marker gene expression, iOPCs showed a stronger potential for astrocytic differentiation and exhibited more significant enrichment in gene sets functionally associated with the negative regulation of OL differentiation. However, since multiple pro-differentiation factors such as T3 are commonly used to induce OL differentiation in vitro, such differences are masked under in vitro conditions and only become evident in cell transplantation experiments.

Transcription factors are a special class of protein molecules that regulate the expression of a large number of genes, characterized by a “small input, large output” effect. SOX10 and OLIG2 are the most central upstream transcription factors for OLs [24,25,26], and they are often used as cellular markers for OPCs. SOX10 belongs to the SOXE family of transcription factors (SOX8/9/10), which act as core and indispensable regulators of neural crest development. In particular, SOX10 governs the specification and differentiation of diverse neural crest derivatives, including melanocytes, the enteric nervous system, Schwann cells, the inner ear, and olfactory ensheathing cells [27]. Activation of OLIG2 is the primary step in the specification of human OPCs [28], whereas SOX10 acts downstream of OLIG2 and participates in both early development and late differentiation of OPCs. However, its role during the formation of early oligodendrocyte progenitor cells (e-OPCs) can be compensated for by SOX8 and SOX9 [26]. NKX2-2 is expressed at a later stage and is generally believed to terminate proliferative signaling by inhibiting PDGFRα, thereby initiating the terminal differentiation program [29]. Therefore, although the normal activation of OLIG2 and functional redundancy of the SOXE family allow the successful induction of NSCs into iOPCs, insufficient activation of SOX10 in iOPCs still compromises their subsequent differentiation potential.

Studies have shown that, as multipotent stem cells, the fate determination stage that affects the terminal differentiation potential of OPCs may begin as early as the induction stage of NSCs [6,30]. We therefore aimed to intervene before NSCs were fully converted into OPCs. Considering that continuous passage under antibiotic selection was still required after lentiviral infection to obtain stable cell lines for subsequent experiments, we finally established the experimental protocol: NSCs were first infected with *SOX10*-carrying lentivirus, followed by induction to generate iOPCs with early *SOX10* overexpression. The expression differences of several marker genes between RNA-seq and RT-qPCR arise from distinctions in the principles, sensitivity, and statistical strategies of the two detection techniques. Moreover, the inconsistency between changes at the RNA and protein levels is related to biological processes, including post-transcriptional regulation, protein translation, and degradation. Overall, although the positive rate and expression level of OPC markers changed to some extent in the experimental iOPCs, their cellular identity as OPCs was preserved, and their survival and proliferation abilities remained unchanged. Migration capacity is an important foundation for OPCs in transplantation therapy; however, the overexpression of *SOX10* significantly inhibited cell migration rates. This may prolong the time required for transplanted cells to migrate to demyelinated lesions, thus limiting the therapeutic efficiency of iOPC-based transplantation. OPCs possess the ability to differentiate into both OL and astrocyte lineages, which is related to OPC heterogeneity and is also influenced by the microenvironment [31,32]. Typical mature OLs display a multilevel, highly branched morphology, and the complexity of cellular branches increases with differentiation and maturation, eventually forming membrane-like structures. According to differentiation status, OLs can be classified into pre-OLs, immature OLs, and mature OLs [33,34]. In the self-differentiation medium without additional pro-differentiation factors, experimental iOPCs differentiated into more mature OLs and fewer astrocytes, and the differentiated OLs exhibited more cellular branches and a more mature phenotype. Nevertheless, the functional myelination capacity of these mature OLs remains to be further verified by in vivo transplantation assays.

Gene transcription is predicated on chromatin accessibility. In human cells, most DNA strands are tightly wrapped around histones to form nucleosomes. During transcription or replication, nucleosomes must disassemble to expose DNA, thereby enabling transcription initiation and the regulation of gene expression. The degree to which cis-regulatory elements (e.g., promoters, enhancers) and trans-acting factors can physically contact chromatin DNA is defined as chromatin accessibility [35]. ATAC-seq is one of the most widely used methods for studying chromatin accessibility and can be applied to identify promoter regions and potential enhancers or silencers [36]. ATAC-seq results have confirmed that TFBSs capable of binding the transcription factor SOX10 are widely present in both NSCs and iOPCs, suggesting that early overexpression of *SOX10* confers extensive gene regulatory potential. Studies have shown that the chromatin of neural progenitor cells is broadly accessible, harboring multiple regulatory regions that maintain pluripotency and proliferative capacity. During subsequent differentiation, chromatin becomes highly focused on the core promoters of lineage-specific functional genes—a classic epigenetic signature of cells exiting pluripotency and undergoing lineage specification [37]. Compared with the control group, the experimental iOPCs exhibited a significantly increased proportion of open regions in core promoter regions (≤1 kb). This indicates that the experimental group drives a shift in chromatin open regions from a diffuse, broad distribution pattern toward concentrated core promoter regions, which is fully consistent with the classic chromatin dynamics observed during stem cell differentiation. In the present study, the chromatin remodeling process driven by experimental iOPCs suggests that, by optimizing the precision of chromatin accessibility, iOPCs are promoted to develop toward a more mature and functionally specialized state.

Although ATAC-seq can analyze changes in genome-wide chromatin accessibility and indicate potential regulatory regions, it fails to clarify the direct interaction between transcription factors and these open regions. Therefore, ChIP-seq using specific antibodies was employed to identify the specific binding sites of transcription factors. In experimental iOPCs, SOX10 mostly bound directly to target gene promoters, whereas in the control group, SOX10 predominantly associated with downstream or distal regulatory elements. This suggests that SOX10 participates in regulation only in an indirect and cooperative manner at low expression levels, while, at high expression levels, it can extensively bind to promoters and directly participate in the regulation of transcription initiation. We also investigated whether there is a causal relationship between increased chromatin accessibility and enhanced SOX10 binding. The results indicated that elevated chromatin openness is not the major direct factor driving the enrichment of SOX10 at promoter regions, further supporting that the upregulated expression of SOX10 is the key cause of its enhanced transcriptional regulation. The number and length of overlapping regions between ATAC-seq and ChIP-seq reflect the correlation between chromatin accessibility and transcription factor binding, which differed significantly between the two iOPC groups. In experimental iOPCs, the overlapping regions were abundant and long, showing a strong correlation between SOX10 binding and chromatin accessibility. These regions were enriched in multiple functional gene sets related to OL differentiation, as well as critical OPC-associated signaling pathways, including the “hsa04150 mTOR signaling pathway”, “hsa04310 Wnt signaling pathway”, “hsa04012 ErbB signaling pathway”, and “hsa04330 Notch signaling pathway” [38,39,40,41], indicating greater functional activity.

SOX10 belongs to the high-mobility group (HMG) box transcription factor family. A key characteristic of this class of proteins is their weak binding affinity to DNA, and they often rely on interactions with other transcription factors to enhance binding specificity. It can be anchored to complexes formed by other transcription factors already bound to target gene promoters via protein–protein interactions, without directly recognizing its own motif [42]. ChIP-seq analysis revealed that 26.54% of target sequences in the experimental iOPCs contained the SOX10-binding motif, and they were significantly enriched for binding sequences of transcription factors, including YY1, ELK4, and ELK1. This suggests that these transcription factors may act cooperatively with SOX10 at the chromatin level. They can function together as protein complexes, or SOX10 binding can trigger chromatin remodeling to create favorable conditions for the subsequent binding of other transcription factors. This phenomenon is commonly reported in other studies; for example, SOX10 forms a heterodimer with PAX3 to mediate the activation of the conserved c-RET enhancer [43], and SOX10 cooperates with MITF to regulate the tumor suppressor DIRC3 [44]. Although the control iOPCs were also enriched for some motifs of other transcription factors, the number was relatively small, and no SOX10 motif was enriched. This indicates that low-expression SOX10 lacks the ability to bind DNA independently and requires the assistance of other factors.

In this study, overexpressed *SOX10* markedly reshaped its chromatin-binding profile and enhanced genome-wide chromatin accessibility, yet it did not trigger large-scale significant changes in the transcriptome. This phenomenon may be associated with the involvement of SOX10 in the poised-state regulatory mechanism [45]. During cellular development and differentiation, multiple key transcription factors can drive target genes into a state characterized by open chromatin and increased transcription factor binding but no immediate significant upregulation of expression—the so-called poised state. Genes in the poised state can be rapidly and synchronously activated upon subsequent stimulation with specific signals [46]. Therefore, as a core transcription factor of the glial lineage, the primary function of SOX10 may not be direct transcriptional activation. Instead, it may prime the transcriptional potential of downstream differentiation genes by reshaping its chromatin-binding pattern. The numerous newly acquired binding targets of SOX10 remain in a poised but inactive state, likely awaiting additional regulatory events after the initiation of differentiation. Upon receiving extracellular differentiation signals and initiating the differentiation program, SOX10 may act cooperatively with other factors to trigger the transcription of its target genes.

Further combined analysis revealed that SOX10 exerts dual regulatory functions on downstream target genes; that is, it acts as a transcriptional activator to promote the expression of *FOS*, *JUN*, *EGR1*, and other genes while also functioning as a transcriptional repressor to inhibit the expression of *CALR*, *MANF*, *HSPA5*, and other genes. These results confirm that SOX10 possesses both transcriptional activation and repression activities in iOPCs, suggesting that it may maintain the homeostatic balance between proliferation and differentiation of iOPCs by precisely regulating the expression of distinct target genes. Functional enrichment analysis of these SOX10 direct target genes further identified *FOS* and *JUN* as the key downstream targets, which serve as convergent nodes of signaling pathways, including the hsa04010 MAPK signaling pathway and hsa04024 cAMP signaling pathway. FOS and JUN are also critical transcription factors that form the AP-1 (Activator Protein-1) complex, which is widely implicated in cell proliferation, differentiation, stress responses, and signaling regulation. Among the genes directly repressed by SOX10, *LHX2* is an important determinant of cell fate. LHX2 cooperates with transcription factors, including DMRT5, PAX6, and NEUROG2, to promote neurogenesis and inhibit gliogenesis [47]. Based on these findings, we propose that the repression of *LHX2* by SOX10 may represent one of the critical molecular mechanisms by which SOX10 regulates the fate transition of OPCs during differentiation.

To broaden the clinical application of this study, we further explored suitable small-molecule drug interventions to enhance the expression of the *SOX10* gene. Through a series of drug screening assays, three small-molecule compounds—TSA, Dabrafenib, and Fedratinib—were ultimately identified, which significantly promoted *SOX10* mRNA expression without compromising the viability of iOPCs. These compounds may serve as promising candidates for optimizing the induction medium formulation for OPCs. Nevertheless, due to the use of HEK293T cells as a model cell line for rapid preliminary high-throughput drug screening, compounds with weak activity in HEK293T cells but specific regulatory effects on iOPCs may still be overlooked. We added these small-molecule drugs during the induction of iOPCs from NSCs, aiming to transiently upregulate SOX10 expression during the induction phase. It was confirmed that elevating SOX10 at the differentiation induction stage also improved the OL differentiation potential of iOPCs. Combined with the results of transgenic experiments, these findings indicate that enhancing SOX10 expression either in NSCs or during iOPC induction can generate iOPCs with improved differentiation capacity. We also observed that the drug-treated iOPCs achieved higher OL differentiation efficiency than those in the transgenic groups. This may be attributed to the sustained overexpression of *SOX10* in the transgenic groups at the late stage of OL maturation, which conversely exerts certain inhibitory effects on OL maturation. This suggests that SOX10 expression requires strict temporal regulation [48].

## 5. Conclusions

Starting from the heterogeneity between OPCs of different origins, this study analyzed the differences in gene expression profiles between iOPCs and fetal OPCs through transcriptomic data analysis. We identified SOX10 as the core upstream transcription factor responsible for the differentiation potential discrepancy between these two OPC populations and verified, through multiple cellular experiments, that SOX10 improves the differentiation efficiency of iOPCs toward the OL lineage. Early overexpression of *SOX10* markedly altered the chromatin accessibility of numerous genes and effectively promoted the lineage specification of iOPCs. Although it did not trigger widespread transcriptomic changes at the OPC stage, its core function was to significantly reshape SOX10’s own binding targets in iOPCs, enhance the priority of direct binding and regulation at promoter regions, and participate in the regulation of functional genes related to glial cell fate determination and OL differentiation. JUN and FOS are important downstream transcription factors in signaling pathways, and they can be directly bound and upregulated by highly expressed SOX10. Finally, three small-molecule drugs—TSA, Dabrafenib, and Fedratinib—that enhance SOX10 expression in iOPCs were screened, providing new options for optimizing OPC induction media. Nevertheless, this study is limited to in vitro experiments, and the therapeutic relevance of our findings requires further functional verification through in vivo transplantation assays.

## Figures and Tables

**Table 1:** Top motif enrichment analysis of upregulated DBRs in experimental iOPCs.

Motif	Name	*p*-Value	*q*-Value (Benjamini)	# Target Sequences with Motif	% of Targets Sequences with Motif
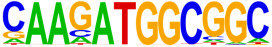	YY1(Zf)	1.00 × 10^−146^	0.0000	744	7.59%
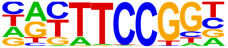	ELK4(ETS)	1.00 × 10^−143^	0.0000	3253	33.21%
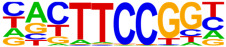	ELK1(ETS)	1.00 × 10^−141^	0.0000	3214	32.81%
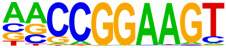	ETS(ETS)	1.00 × 10^−133^	0.0000	1881	19.20%
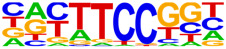	FLI1(ETS)	1.00 × 10^−131^	0.0000	3998	40.81%

## Data Availability

The raw ATAC-seq, ChIP-seq, and RNA-seq data generated in this study have been deposited in the GSA-human database under accession number HRA017540 and will be made available upon reasonable request.

## References

[1] Akay L.A., Effenberger A.H., Tsai L.H. (2021). Cell of all trades: Oligodendrocyte precursor cells in synaptic, vascular, and immune function. Genes Dev..

[2] Figarella-Branger D., Colin C., Baeza-Kallee N., Tchoghandjian A. (2022). A2B5 Expression in Central Nervous System and Gliomas. Int. J. Mol. Sci..

[3] Van Tilborg E., De Theije C.G.M., Van Hal M., Wagenaar N., De Vries L.S., Benders M.J., Rowitch D.H., Nijboer C.H. (2018). Origin and dynamics of oligodendrocytes in the developing brain: Implications for perinatal white matter injury. Glia.

[4] Sim F.J., Mcclain C.R., Schanz S.J., Protack T.L., Windrem M.S., Goldman S.A. (2011). CD140a identifies a population of highly myelinogenic, migration-competent and efficiently engrafting human oligodendrocyte progenitor cells. Nat. Biotechnol..

[5] Ye D., Wang Q., Yang Y., Chen B., Zhang F., Wang Z., Luan Z. (2023). Identifying Genes that Affect Differentiation of Human Neural Stem Cells and Myelination of Mature Oligodendrocytes. Cell Mol. Neurobiol..

[6] Namchaiw P., Wen H., Mayrhofer F., Chechneva O., Biswas S., Deng W. (2019). Temporal and partial inhibition of GLI1 in neural stem cells (NSCs) results in the early maturation of NSC derived oligodendrocytes in vitro. Stem Cell Res. Ther..

[7] Chamling X., Kallman A., Fang W., Berlinicke C.A., Mertz J.L., Devkota P., Pantoja I.E.M., Smith M.D., Ji Z., Chang C. (2021). Single-cell transcriptomic reveals molecular diversity and developmental heterogeneity of human stem cell-derived oligodendrocyte lineage cells. Nat. Commun..

[8] Jakovcevski I., Filipovic R., Mo Z., Rakic S., Zecevic N. (2009). Oligodendrocyte development and the onset of myelination in the human fetal brain. Front. Neuroanat..

[9] Seeker L.A., Williams A. (2022). Oligodendroglia heterogeneity in the human central nervous system. Acta Neuropathol..

[10] Zheng J., Chen Y., Hu Y., Zhu Y., Lin J., Xu M., Zhang Y., Song W., Chen X. (2025). Lineage trajectories and fate determinants of postnatal neural stem cells and ependymal cells in the developing ventricular zone. PLoS Biol..

[11] Arshadi C., Günther U., Eddison M., Harrington K.I.S., Ferreira T.A. (2021). SNT: A unifying toolbox for quantification of neuronal anatomy. Nat. Methods.

[12] Yu G. (2024). Thirteen years of clusterProfiler. Innovation.

[13] Wang Q., Li M., Wu T., Zhan L., Li L., Chen M., Xie W., Xie Z., Hu E., Xu S. (2022). Exploring Epigenomic Datasets by ChIPseeker. Curr. Protoc..

[14] Young M.D., Wakefield M.J., Smyth G.K., Oshlack A. (2010). Gene ontology analysis for RNA-seq: Accounting for selection bias. Genome Biol..

[15] Bu D., Luo H., Huo P., Wang Z., Zhang S., He Z., Wu Y., Zhao L., Liu J., Guo J. (2021). KOBAS-i: Intelligent prioritization and exploratory visualization of biological functions for gene enrichment analysis. Nucleic Acids Res..

[16] Pol Su O.B.M., Vedia B., Sim F. Transcription Profile of Fetal Human CD140a-Defined OPC Differentiation In Vitro [DB]. GEO(GSE36431), 2016. https://www.ncbi.nlm.nih.gov/geo/query/acc.cgi?acc=GSE36431.

[17] Cheli V.T., Santiago González D.A., Marziali L.N., Zamora N.N., Guitart M.E., Spreuer V., Pasquini J.M., Paez P.M. (2018). The Divalent Metal Transporter 1 (DMT1) Is Required for Iron Uptake and Normal Development of Oligodendrocyte Progenitor Cells. J. Neurosci..

[18] Balmer N.V., Klima S., Rempel E., Ivanova V.N., Kolde R., Weng M.K., Meganathan K., Henry M., Sachinidis A., Berthold M.R. (2014). From transient transcriptome responses to disturbed neurodevelopment: Role of histone acetylation and methylation as epigenetic switch between reversible and irreversible drug effects. Arch. Toxicol..

[19] Krug A.K., Kolde R., Gaspar J.A., Rempel E., Balmer N.V., Meganathan K., Vojnits K., Baquié M., Waldmann T., Ensenat-Waser R. (2013). Human embryonic stem cell-derived test systems for developmental neurotoxicity: A transcriptomics approach. Arch. Toxicol..

[20] King A.J., Arnone M.R., Bleam M.R., Moss K.G., Yang J., Fedorowicz K.E., Smitheman K.N., Erhardt J.A., Hughes-Earle A., Kane-Carson L.S. (2013). Dabrafenib; preclinical characterization, increased efficacy when combined with trametinib, while BRAF/MEK tool combination reduced skin lesions. PLoS ONE.

[21] Hao Y., Chapuy B., Monti S., Sun H.H., Rodig S.J., Shipp M.A. (2014). Selective JAK2 inhibition specifically decreases Hodgkin lymphoma and mediastinal large B-cell lymphoma growth in vitro and in vivo. Clin. Cancer Res..

[22] Stallcup W.B. (1981). The NG2 antigen, a putative lineage marker: Immunofluorescent localization in primary cultures of rat brain. Dev. Biol..

[23] Bechler M.E., Byrne L., Ffrench-Constant C. (2015). CNS Myelin Sheath Lengths Are an Intrinsic Property of Oligodendrocytes. Curr. Biol..

[24] Sock E., Wegner M. (2021). Using the lineage determinants Olig2 and Sox10 to explore transcriptional regulation of oligodendrocyte development. Dev. Neurobiol..

[25] Reiprich S., Wegner M. (2015). From CNS stem cells to neurons and glia: Sox for everyone. Cell Tissue Res..

[26] Hines J.H. (2021). Evolutionary Origins of the Oligodendrocyte Cell Type and Adaptive Myelination. Front. Neurosci..

[27] Schock E.N., Labonne C. (2020). Sorting Sox: Diverse Roles for Sox Transcription Factors During Neural Crest and Craniofacial Development. Front. Physiol..

[28] Hu B.Y., Du Z.W., Li X.J., Ayala M., Zhang S.C. (2009). Human oligodendrocytes from embryonic stem cells: Conserved SHH signaling networks and divergent FGF effects. Development.

[29] Zhu Q., Zhao X., Zheng K., Li H., Huang H., Zhang Z., Mastracci T., Wegner M., Chen Y., Sussel L. (2014). Genetic evidence that Nkx2.2 and Pdgfra are major determinants of the timing of oligodendrocyte differentiation in the developing CNS. Development.

[30] Hu B.Y., Du Z.W., Zhang S.C. (2009). Differentiation of human oligodendrocytes from pluripotent stem cells. Nat. Protoc..

[31] Raff M.C., Miller R.H., Noble M. (1983). A glial progenitor cell that develops in vitro into an astrocyte or an oligodendrocyte depending on culture medium. Nature.

[32] Hu J.G., Lü H.Z., Wang Y.X., Bao M.S., Zhao B.M., Zhou J.S. (2010). BMP signaling mediates astrocyte differentiation of oligodendrocyte progenitor cells. Tohoku J. Exp. Med..

[33] Sajad M., Zahoor I., Rashid F., Cerghet M., Rattan R., Giri S. (2024). Pyruvate Dehydrogenase-Dependent Metabolic Programming Affects the Oligodendrocyte Maturation and Remyelination. Mol. Neurobiol..

[34] Li H., Chen Y., Niu J., Yi C. (2022). New insights into the immunologic role of oligodendrocyte lineage cells in demyelination diseases. J. Biomed. Res..

[35] Klemm S.L., Shipony Z., Greenleaf W.J. (2019). Chromatin accessibility and the regulatory epigenome. Nat. Rev. Genet..

[36] Buenrostro J.D., Giresi P.G., Zaba L.C., Chang H.Y., Greenleaf W.J. (2013). Transposition of native chromatin for fast and sensitive epigenomic profiling of open chromatin, DNA-binding proteins and nucleosome position. Nat. Methods.

[37] Rhodes C.T., Thompson J.J., Mitra A., Asokumar D., Lee D.R., Lee D.J., Zhang Y., Jason E., Dale R.K., Rocha P.P. (2022). An epigenome atlas of neural progenitors within the embryonic mouse forebrain. Nat. Commun..

[38] Hu X., Xiao G., He L., Niu X., Li H., Lou T., Hu Q., Yang Y., Xu Q., Wei Z. (2021). Sustained ErbB Activation Causes Demyelination and Hypomyelination by Driving Necroptosis of Mature Oligodendrocytes and Apoptosis of Oligodendrocyte Precursor Cells. J. Neurosci..

[39] Ornelas I.M., Khandker L., Wahl S.E., Hashimoto H., Macklin W.B., Wood T.L. (2020). The mechanistic target of rapamycin pathway downregulates bone morphogenetic protein signaling to promote oligodendrocyte differentiation. Glia.

[40] Niu J., Yu G., Wang X., Xia W., Wang Y., Hoi K.K., Mei F., Xiao L., Chan J.R., Fancy S.P.J. (2021). Oligodendroglial ring finger protein Rnf43 is an essential injury-specific regulator of oligodendrocyte maturation. Neuron.

[41] Park H.C., Appel B. (2003). Delta-Notch signaling regulates oligodendrocyte specification. Development.

[42] Wissmüller S., Kosian T., Wolf M., Finzsch M., Wegner M. (2006). The high-mobility-group domain of Sox proteins interacts with DNA-binding domains of many transcription factors. Nucleic Acids Res..

[43] Lang D., Epstein J.A. (2003). Sox10 and Pax3 physically interact to mediate activation of a conserved c-RET enhancer. Hum. Mol. Genet..

[44] Coe E.A., Tan J.Y., Shapiro M., Louphrasitthiphol P., Bassett A.R., Marques A.C., Goding C.R., Vance K.W. (2019). The MITF-SOX10 regulated long non-coding RNA DIRC3 is a melanoma tumour suppressor. PLoS Genet..

[45] Bernstein B.E., Mikkelsen T.S., Xie X., Kamal M., Huebert D.J., Cuff J., Fry B., Meissner A., Wernig M., Plath K. (2006). A bivalent chromatin structure marks key developmental genes in embryonic stem cells. Cell.

[46] Gaertner B., Johnston J., Chen K., Wallaschek N., Paulson A., Garruss A.S., Gaudenz K., De Kumar B., Krumlauf R., Zeitlinger J. (2012). Poised RNA polymerase II changes over developmental time and prepares genes for future expression. Cell Rep..

[47] Muralidharan B., Keruzore M., Pradhan S.J., Roy B., Shetty A.S., Kinare V., D’souza L., Maheshwari U., Karmodiya K., Suresh A. (2017). Dmrt5, a Novel Neurogenic Factor, Reciprocally Regulates Lhx2 to Control the Neuron-Glia Cell-Fate Switch in the Developing Hippocampus. J. Neurosci..

[48] Aprato J., Sock E., Weider M., Elsesser O., Fröb F., Wegner M. (2020). Myrf guides target gene selection of transcription factor Sox10 during oligodendroglial development. Nucleic Acids Res..

